# IL‐34 in hepatoblastoma cells potentially promote tumor progression via autocrine and paracrine mechanisms

**DOI:** 10.1002/cam4.4537

**Published:** 2022-02-08

**Authors:** Tomoaki Irie, Daiki Yoshii, Yoshihiro Komohara, Yukio Fujiwara, Masashi Kadohisa, Masaki Honda, Shinya Suzu, Toshiharu Matsuura, Kenichi Kohashi, Yoshinao Oda, Taizo Hibi

**Affiliations:** ^1^ Department of Cell Pathology Graduate School of Medical Sciences Kumamoto University Kumamoto Japan; ^2^ Department of Pediatric Surgery and Transplantation Graduate School of Medical Sciences Kumamoto University Kumamoto Japan; ^3^ Center for Metabolic Regulation of Healthy Aging Kumamoto University Kumamoto Japan; ^4^ Joint Research Center for Human Retrovirus Infection Kumamoto University Kumamoto Japan; ^5^ Department of Pediatric Surgery Graduate School of Medical Sciences Kyushu University Fukuoka Japan; ^6^ Department of Anatomic Pathology Graduate School of Medical Sciences Kyushu University Fukuoka Japan

**Keywords:** embryonal, fetal, hepatoblastoma, IL‐34, macrophage

## Abstract

Hepatoblastoma is the most common pediatric liver tumor, but little research has been done on the role of macrophages in hepatoblastoma. The purpose of this study was to gain insight into potential roles for macrophages in hepatoblastoma. Paraffin‐embedded specimens from 56 patients who underwent surgical resection were examined with immunohistochemical staining for the macrophage‐specific markers, Iba1 and CD163. Significant differences were seen among histological subtypes. Significantly increased numbers of macrophages were detected in embryonal components compared to fetal components in the mixed epithelial type. In vitro studies using human monocyte‐derived macrophages and two hepatoblastoma cell lines (HepG2 and Huh6) were performed. Conditioned medium from these cell lines induced increased CD163 expression in macrophages. Direct co‐culture with macrophages induced tumor cell proliferation via induction of protumor cytokine secretion from macrophages. Direct co‐culture with macrophages also induced interleukin (IL)‐34 overexpression by Huh6 cells via Brd4 signaling. IL‐34 overexpression promoted tumor cell proliferation and chemoresistance. High IL‐34 and Brd4 expression was detected in embryonal components, which have potentially higher proliferation activity than fetal components. In conclusion, IL‐34 expression in embryonal components may induce macrophage chemotaxis in a paracrine manner, and tumor cell proliferation and chemoresistance in an autocrine manner. IL‐34 is a potential therapeutic target for hepatoblastoma.

## Graphical abstract

Fetal component of hepatoblatoma secret IL34 which promote tumor proliferation and chemo‐resistance by autocrine manner. IL‐34 also potentially induce protumor chemotaxis in fetal component.

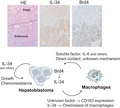

## 1 INTRODUCTION

Hepatoblastoma is a common malignant pediatric liver cancer, with an incidence of 1.5 cases/million people per year.^1^, ^2^ Advances in chemotherapy and surgical techniques have improved the survival rate to up to 70–80% over the recent decades. However, survival of patients with metastatic disease remains unsatisfactory.^3^, ^4^ Hepatoblastoma is pathologically classified by the International Pediatric Liver Tumor Consensus Classification as epithelial, mixed epithelial, and mesenchymal, and the main subtypes are fetal, mixed fetal, embryonal, mesenchymal, and mixed epithelial.^5^, ^6^ However, the association between the histological classification and clinical impact is unclear.

In solid tumors, many non‐tumor host cells such as inflammatory leukocytes, endothelial cells, and fibroblasts are present in the tumor microenvironment (TME). Macrophages that infiltrate in tumor tissues are referred to as tumor‐associated macrophages (TAMs).^7^, ^8^, ^9^ Recent studies have shown that TAMs play critical roles in multiple aspects, and contribute to tumor invasion, growth, therapeutic resistance, and metastasis by producing various mediators in many tumors.^10^, ^11^ Therefore, targeting the differentiation or chemotaxis of TAMs in the TME has been suggested as a novel anticancer strategy.

Many retrospective studies using paraffin sections and immunohistochemistry (IHC) of macrophages have been published. These studies reported that an increased density of CD163‐expressing TAMs predicted a worse clinical course in solid tumors in adults.^12^, ^13^ In vitro studies using human tumor cells and macrophages, as well as animal studies, have demonstrated the protumor functions of CD163‐positive TAMs.^14^, ^15^, ^16^ In pediatric cancer, Hashimoto et al. reported that a high density of CD163‐positive TAMs is correlated with poor prognosis in neuroblastoma.^17^ This group also suggested that cell‐cell interactions between fibroblast and TAMs support neuroblastoma progression. Because the function and significance of TAMs in hepatoblastoma have not been clarified, we investigated the impact of TAMs in hepatoblastoma subtypes and on tumor cell growth using paraffin‐embedded samples and cell culture studies.

## 2 MATERIALS AND METHODS

### 2.1 Samples

From January 2000 to March 2020, 56 hepatoblastoma patients underwent surgical resection followed by pathological confirmation at the Kumamoto University Hospital (Kumamoto, Japan) or Kyushu University Hospital (Fukuoka, Japan). Informed consent for this study was obtained from all patients, and study designs and protocols were approved by the Kumamoto University (#2224) and Kyushu University (#2020‐660) Review Boards. Staging was assessed according to the PRETEXT system designed by the International Childhood Liver Tumor Strategy Group (SIOPEL). Patients’ characteristics are summarized in Table 1.

**TABLE 1:** Patients’ characteristics

Age (year)	*n* = 56	%
<12 months	11	19.6
1–3	35	62.5
4–9	6	10.7
10<	4	7.1
Sex		
Male	29	51.8
Female	27	48.2
PRETEXT stage		
Ⅰ	4	7.1
Ⅱ	15	26.8
Ⅲ	19	33.9
Ⅳ	18	32.1
AFP (ng/ml)		
<100	0	0
100–10,000	5	8.9
10,000–1,000,000	40	71.4
1,000,000<	11	19.6
Annotation factors		
V	5	8.9
P	12	21.4
R	1	1.8
M	9	16.1
None	29	51.8
Recurrence		
Yes	13	23.2
No	43	76.8
Histology		
Fetal	20	35.7
Embryonal	5	8.9
Mixed epitherial	21	37.5
MEM	9	16.1
Macrotrabecular	1	1.8
Small cell undifferentiated	0	0

### 2.2 Immunohistochemistry (IHC)

Tissue samples were fixed in 10% neutral buffered formalin and embedded in paraffin. The specimens were cut at a thickness of 3 μm from paraffin‐embedded conventional blocks and deparaffinized with xylene and ethanol. Hepatoblastoma foci were stained to detect the expression of Ki‐67 (mouse monoclonal; M7240, DAKO), Iba1 (rabbit polyclonal; 019‐19741, WAKO), CD163 (mouse monoclonal; AM3K, Transgenic, Kumamoto, Japan), SALL4 (mouse monoclonal; Abcam, Cambridge, UK, ab57577), bromodomain‐containing protein 4 (Brd4, rabbit monoclonal; Abcam, ab128874), and interleukin (IL)‐34 (mouse monoclonal; 1D12, Abcam). Horseradish peroxidase‐labeled goat anti‐rabbit or anti‐mouse antibodies (Nichirei) were used as secondary antibodies.

### 2.3 Cell culture of macrophages

Human monocyte‐derived macrophages (HMDMs) were obtained from healthy donors in accordance with protocols approved by the Kumamoto University Hospital Review Board (#1169). Monocytes were isolated by peripheral blood of healthy volunteer donors using the RosetteSep Human Monocyte Enrichment Cocktail (STEMCELL Technologies). These isolated monocytes were plated in UpCELL 6‐well plates (2 × 10^5^ cells/well; CellSeed, Tokyo, Japan) and cultured in AIM‐V medium (Thermo Fisher) supplemented with macrophage‐colony stimulating factor (100 ng/ml, M‐CSF, WAKO), granulocyte macrophage‐colony stimulating factor (1 ng/ml, WAKO), and 2% human serum for 7 days to induce differentiation of macrophages.

### 2.4 Cell‐ELISA

CD163 expression on human monocyte‐derived macrophages (HMDMs) was evaluated using a Cell‐ELISA as described previously.^18^ After cells were fixed with paraformaldehyde, each well of a 96‐well plate was blocked with 1% bovine serum albumin (Sigma‐Aldrich). The wells were then incubated with anti‐CD163 antibody AM‐3K (Transgenic, Kumamoto, Japan) 2 µg/ml for 1 h. Thereafter, wells were washed by washing buffer and reacted with HRP‐conjugated anti‐mouse IgG antibody, followed by a reaction with ULTRASENSITIVE TMB (Cosmo Bio). The reaction was stopped by the addition of 1 M sulfuric acid, and the absorbance at 450 nm was read by a micro‐plate reader.

### 2.5 Cell culture of cancer cell lines

Huh6 and HepG2 cells were obtained from JCB Cell Bank. Cells were cultured in D‐MEM/Ham's F‐12 (WAKO) supplemented with 10% fetal bovine serum, 1% minimal essential medium non‐essential amino acids, and 1% penicillin/streptomycin. Mycoplasma infection was routinely checked with a PCR Mycoplasma Detection Set (TAKAYA). Co‐culture of cell lines was performed using 96‐well plates as described previously, and BrdU incorporation was assessed with a BrdU ELISA Kit (Roche, Mannheim, Germany). IL‐34 gene‐coding pcDNA vector^19^ and control vector were transfected using HilyMax transfection reagent (Dojindo). Trans‐well cell culture inset (Thermo Fisher Scientific) was used for indirect co‐culture study.

### 2.6 Cell proliferation assay

Briefly, tumor cells were cultured in a 96‐well plate in quadruplicate before treatment. HMDMs and Huh6 cells were directly cocultured and cell proliferation was tested by BrdU incorporation assay kit (Roche) or cell counting under microscopy. Anti‐BrdU antibody (Santa Cruz Biotech) and anti‐CD204 antibody (clone SRA‐E5, Cosmo Bio) were used for immunocytostaining after BrdU assay as described previously.^20^

### 2.7 Real time quantitative‐PCR

RNA extraction from culture cells and PCR were performed as described previously.^20^ The following primers were used: IL‐10, forward 5'‐GGTTGCCAAGCCTTGTCTGA‐3’ and reverse 5'‐AGGGAGTTCACATGCGCCT‐3’; IL‐6, forward 5'‐ATGTGTGAAAGCAGCAAAGAGG‐3’ and reverse 5'‐GTGATGATTTTCACCAGGCAAG‐3’; IL‐34, forward 5'‐TGTTCAGAATCGCCAACGTC‐3’ and reverse 5'‐GCTCACCAAGACCCACAGATAC‐3’; M‐CSF, forward 5'‐GCTGAAGAGCTGCTTCACCAA‐3’ and reverse 5'‐CATTCTTGACCTTCTCCAGCAA‐3’; CCL2, forward 5'‐CATAGCAGCCACCTTCATTCC‐3’ and reverse 5'‐TGCACTGAGATCTTCCTATTGGTG‐3’; and b‐actin, forward 5'‐ATTCCTATGTGGGCGACGAG‐3’ and reverse 5'‐AAGGTGTGGTGCCAGATTTTC‐3'.

### 2.8 Statistical analysis

Statistical analysis was carried out using StatMate (ATOMS) and JMP7 software (SAS Institute). For all analyses, *p* < 0.05 was considered statistically significant.

## 3 RESULTS

### 3.1 A high density of Iba1‐ and CD163‐positive TAMs was detected in embryonal components in hepatoblastoma

**FIGURE 1:**
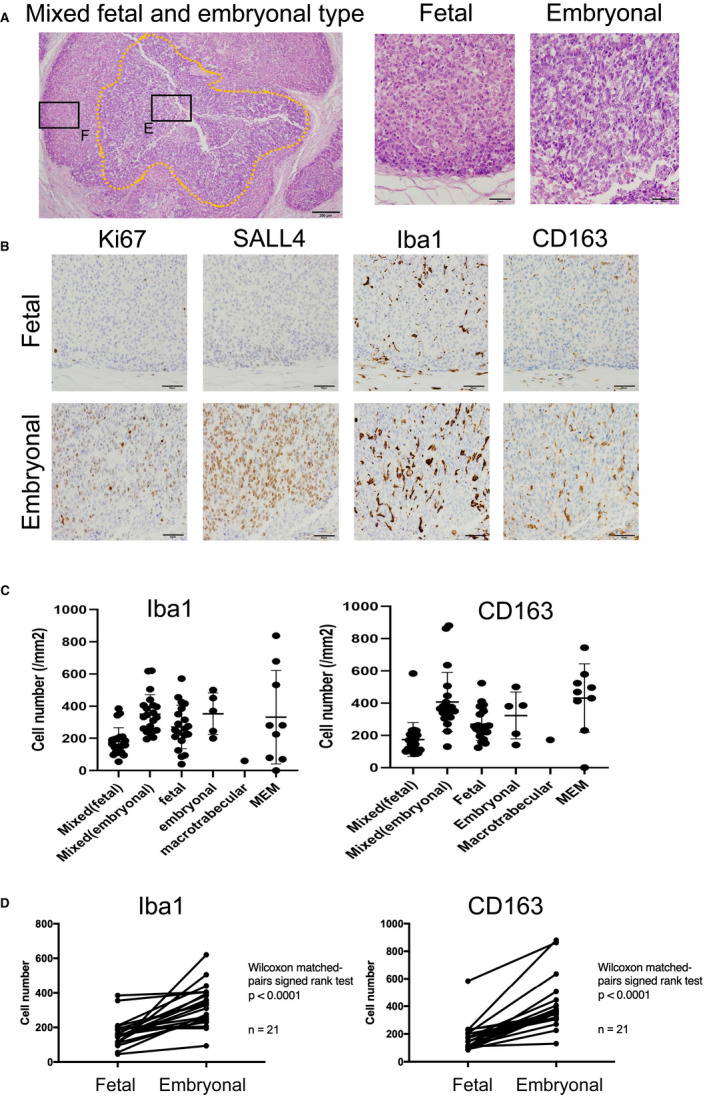
Macrophage distribution in hepatoblastoma cases. (A) Hematoxylin and eosin staining of a hepatoblastoma sample (mixed fetal and embryonal type) is presented. Embryonal component cells have more enlarged nuclei with coarse chromatin and a higher nuclear/cytoplasmic ratio than fetal component cells. (B) Immunohistochemistry (IHC) for Ki‐67, SALL4, Iba1, and CD163 in the mixed fetal and embryonal type. (C) Dot plots of the densities of Iba1‐ and CD163‐positive macrophages among historical types are shown. (D) Iba1‐ and CD163‐positive macrophages were counted in the fetal and embryonal components in the same cases with the mixed fetal and embryonal type (*n* = 21)

First, IHC for Iba1 and CD163 was performed in all cases (*n* = 56), and the cell densities in the tumor area were evaluated. No significant differences in the densities of TAMs were seen between histological subtypes. However, higher densities of TAMs were apparent in embryonal components compared to fetal components in the mixed fetal and embryonal subtype (Figure 1A–C). The densities of TAMs were compared between fetal components and embryonal components in the same cases (*n* = 21, mixed fetal and embryonal subtype), and significantly increased Iba1‐positive and CD163‐positive TAMs were seen in the embryonal components (Figure 1C).

Hepatoblastoma embryonal type tumor cells had enlarged nuclei, and the cytoplasm was dark and granular compared with fetal type cells (Figure 1A) and positive for SALL4 and Ki67 in consistent with previous reports^21^, ^22^ (Figure 1B). These data suggest that tumor cells in embryonal components have a higher malignant or aggressive potential than those in fetal components. We hypothesized that cell‐cell interactions between TAMs and tumor cells activated tumor cell proliferation.

### 3.2 Cell‐cell interactions between macrophages and cancer cells induced macrophage M2 polarization and cancer cell proliferation

**FIGURE 2:**
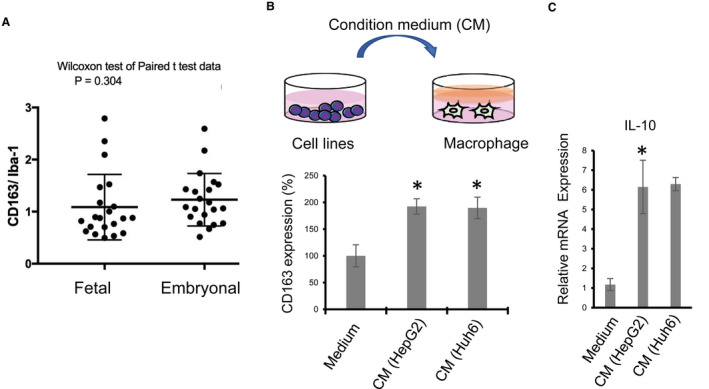
Macrophage activation and hepatoblastoma. (A) CD163‐positive cell ratio among Iba1‐positive macrophages in fetal and embryonal components in the same cases with the mixed fetal and embryonal type (*n* = 21). Human monocyte‐derived macrophages (HMDMs) were stimulated with conditioned medium (CM) from HepG2 and Huh6 cells for 24 h, and CD163 expression and IL‐10 expression were tested with a cell‐ELISA (B) and real‐time PCR (C). *p‐value <0.05 by the Student's *t*‐test

CD163 and IL‐10 are well‐known markers for the M2‐like/protumor phenotype of macrophages.^18^ The ratio of CD163‐positive cells in Iba1‐positive cells indicated the ratio of the M2‐like phenotype of TAMs. When the M2 ratio of TAMs was compared between fetal areas and embryonal areas, no significant difference was observed (Figure 2A). However, most infiltrated TAMs were the M2‐like phenotype both in fetal areas and embryonal areas. We next tested if conditioned medium (CM) from hepatoblastoma cell lines induced M2‐like activation of HMDMs. CD163 and IL‐10 expression was significantly elevated by CM from hepatoblastoma cell lines (Figure 2B, C). Based on these observations, we hypothesized that TAMs are polarized into the M2‐like phenotype, which in turn, stimulates hepatoblastoma cell proliferation.

**FIGURE 3:**
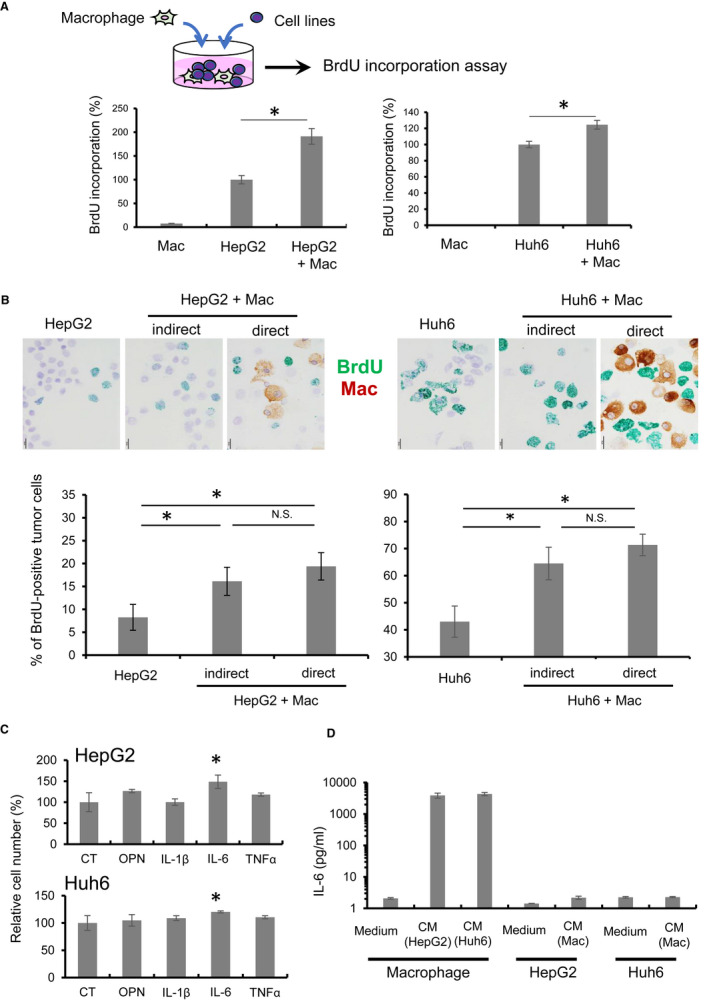
Cell‐cell interactions between macrophages and hepatoblastoma cell lines. (A) Direct co‐culture of HMDMs and cell lines was done in 96‐well plates, and BrdU incorporation in cell lines was tested with a BrdU‐ELISA kit. (B) Indirect and direct co‐culture of HMDMs and cell lines were performed in 6‐well culture plate under low FBS (2%) condition and immunocytostaining were performed to evaluate the BrdU incorporation in cancer cells which were negative for CD204 (a marker for HMDMs). (C) Cell lines were stimulated with osteopontin (OPN), IL‐1β, IL‐6, and tumor necrosis factor (TNF)‐α at a concentration of 10 ng/ml for 24 h, and the cell viability was evaluated with the WST assay. (D) Macrophages were stimulated with CM from cell lines, and each cell line was stimulated with CM from macrophages for 24 h. Then, IL‐6 secretion in medium was evaluated. *p‐value <0.05 by the Student's *t*‐test. N.S.; not significant

To test our hypothesis, we next performed cell culture studies using macrophages and cell lines. Direct co‐culture assays were also performed, and BrdU incorporation into cancer cells was significantly increased by direct interaction with macrophages (Figure 3A). Indirect co‐culture assay using trans‐well culture insert also increased BrdU incorporation into cancer cells (Figure 3B). Although BrdU incorporation seemed to be higher in direct co‐culture cells than indirect co‐culture cells, the difference was not statistically significant (Figure 3B). Next, we investigated macrophage‐derived growth factors that affect hepatoblastoma cell lines. Because osteopontin (OPN), IL‐1β, tumor necrosis factor (TNF)‐α, and IL‐6 are well‐known growth factors derived from macrophages, cancer cells were stimulated with these recombinant proteins, and cell proliferation was assessed with the WST assay. IL‐6 was significantly induced cell proliferation in hepatoblastoma cell lines (Figure 3C). Unstimulated macrophages and hepatoblastoma cell lines secreted low levels of IL‐6. However, notable induction of IL‐6 was induced by stimulation with CM from hepatoblastoma cell lines (Figure 3D).

### 3.3 IL‐34 was a candidate for macrophage chemotaxis into embryonal component

Then unknown chemotactic factors were suggested to be involved in TAM infiltration into embryonal component in mixed fetal and embryonal subtype. IL‐34, M‐CSF and CCL2 were at first listed as factors related to macrophage chemotaxis, and their expression in HMDM and cancer cell lines were tested by real‐time PCR. IL‐34 expression was detected in cancer cell lines but not in HMDMs, whereas M‐CSF and CCL2 expression was seen in HMDMs but not or rarely in cancer cell lines (Figure 4A). IL‐34 mRNA expression was higher in HepG2 than Huh6 cells (Figure 4A). IL‐34 protein expression was detected by IHC, and the result showed strong positive signal was seen in HepG2 but no or very weak signal was observed in Huh6 (Figure 4B).

**IGURE 4:**
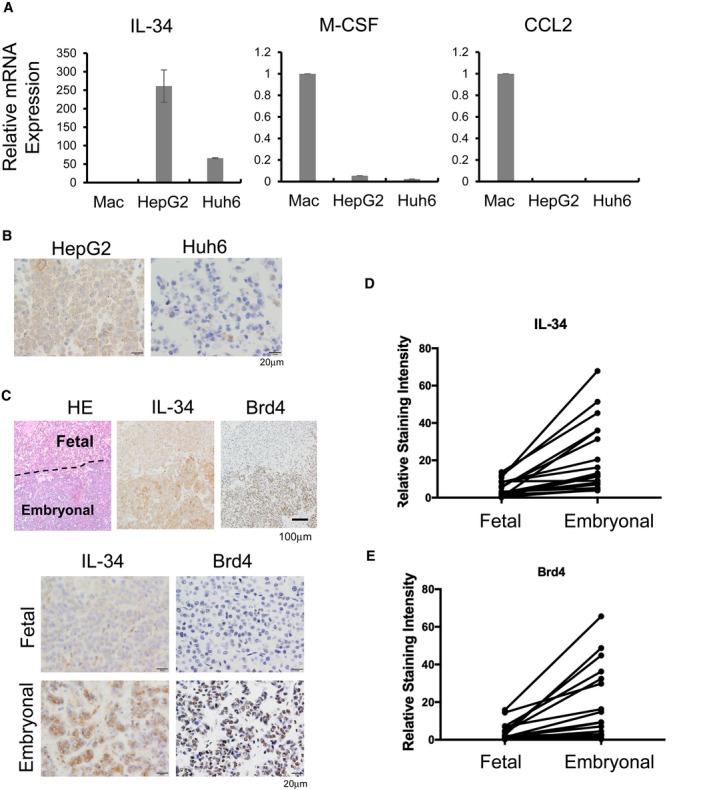
IL‐34 expression in embryonal components. (A) Relative mRNA expression levels of macrophage chemotactic factors (IL‐34, M‐CSF, CCL2) in macrophages (Mac), HepG2 cells, and Huh6 cells were tested with real‐time PCR. (B) IL‐34 expression in HepG2 and Huh6 cell lines was tested with IHC using cell block specimens. (C) Representative IHC for IL‐34 and Brd4 in the border area of fetal and embryonal components. IL‐34‐ (D) and Brd4‐ (E) positive staining in tumor cells was evaluated with Image J software and compared between fetal and embryonal components in the same cases with the mixed fetal and embryonal type (*n* = 21). Wilcoxon paired t‐test was performed (*p* < 0.001)

IHC for IL‐34 was subsequently performed in 21 cases of the mixed fetal and embryonal subtype. Notably, strong IL‐34‐positive signals were seen in embryonal tumor cells, but no or weak signals were detected in fetal tumor cells (Figure 4C, D).

A previous study revealed that IL‐34 production was triggered by Brd4 binding to the IL‐34 promoter,^23^ and therefore, we next tested Brd4 expression. As shown in Figure 4C, E, Brd4 expression was significantly higher in embryonal lesions compared with fetal lesions.

### 3.4 Direct co‐culture with macrophages induced IL‐34 expression in Huh6 cells

To investigate the mechanism of IL‐34 overexpression in tumor cells, we next stimulated Huh6 cells with several cytokines that are considered to be secreted from macrophages. TNF‐α, OPN, IL‐6, and IL‐1β were individually added to Huh6 cell culture, but none of them induced IL‐34 overexpression (data not shown). We next tested whether direct cell‐cell interactions between macrophages and cancer cells induced cancer cell activation by testing if direct co‐culture with macrophages influenced IL‐34 expression in cancer cells. When Huh6 cells were co‐cultured with HMDMs directly, IL‐34 expression in Huh6 cells was significantly elevated by co‐culture (Figure 5A). IL‐34 expression was not induced in HMDMs by co‐culture. CM of macrophages did not affect IL‐34 expression in cancer cells (data not shown). Brd4 expression in Huh6 cells was also significantly increased by co‐culture with HMDMs (Figure 5B).

**IGURE 5:**
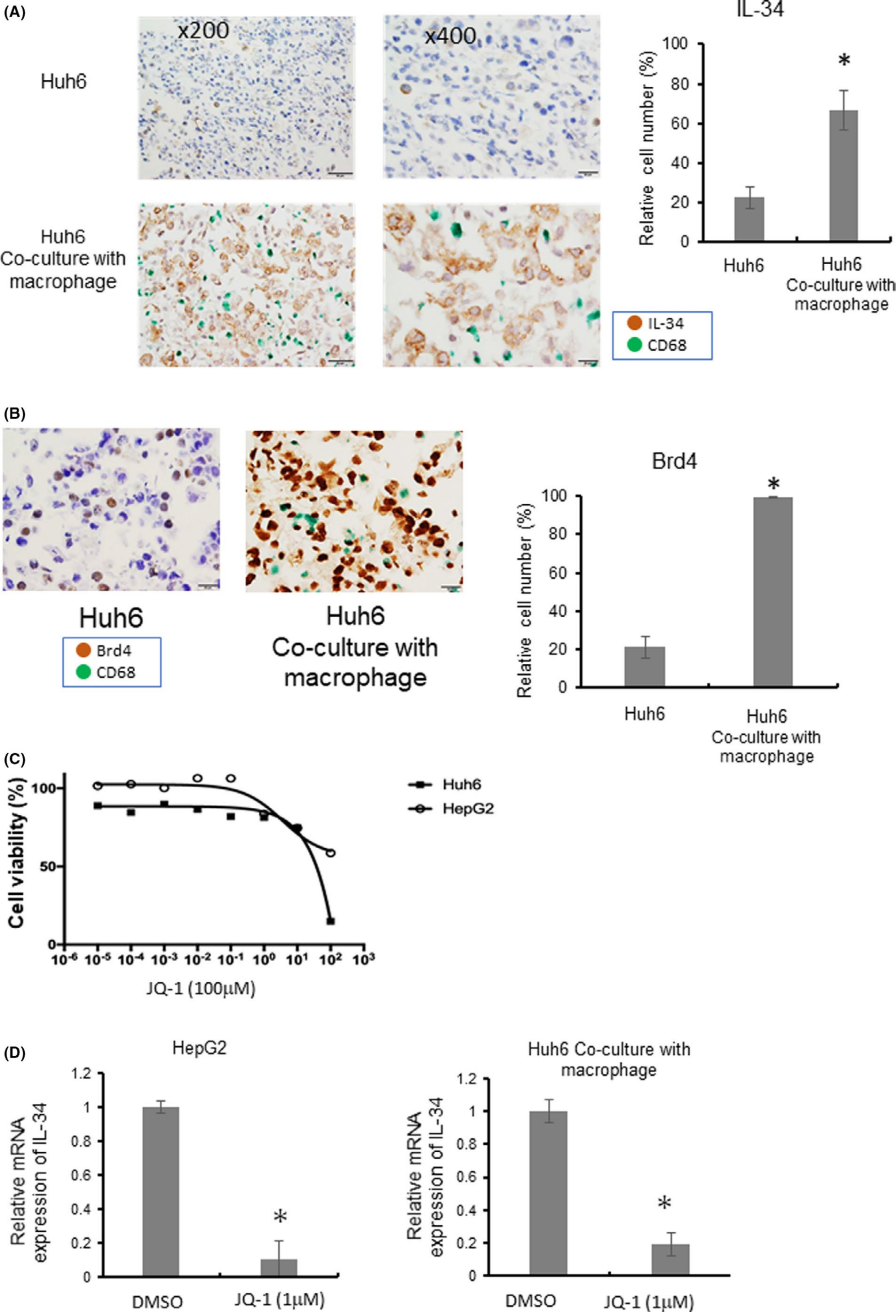
Co‐culture study of hepatoblastoma cell lines and HMDMs. (A) Huh6 cells were cultured directly with or without HMDMs for 24 h, and then cells were fixed in paraformaldehyde, and paraffin‐embedded cell block specimens were prepared. Sections were stained with an anti‐IL‐34 antibody and anti‐CD68 antibody. IL‐34 and CD68 signals were colored with brown and green, respectively. IL‐34‐positive cells (%) among CD68‐negative Huh6 cells were evaluated with microscopy (*n* = 3 each). (B) Brd4 and CD68 signals were colored with brown and green, respectively, and Brd4‐positive cells (%) in Huh6 cells were evaluated with microscopy (*n* = 3 each). (C) HepG2 cells and Huh6 cells were cultured with JQ‐1 for 24 h, and cell viability was assessed with the WST assay. (D) HepG2 cells without co‐culture and Huh6 cells co‐cultured with HMDMs were stimulated with JQ‐1 for 24 h, and mRNA expression of IL‐34 was evaluated with real‐time PCR. *p‐value <0.05 by the Student's *t*‐test

We next examined if IL‐34 in hepatoblastoma cells was regulated by Brd4 by using the Brd4 inhibitor, JQ‐1. Because HepG2 cells express high levels of IL‐34, HepG2 cells were used, and the sensitivity of HepG2 cells against JQ‐1 was examined. HepG2 cell viability was reduced by JQ‐1, but the IC50 was more than 10 nM (Figure 5C). When HepG2 cells were cultured with 1 nM JQ‐1, IL‐34 expression was significantly suppressed (Figure 5D). IL‐34 expression in co‐cultured Huh6 cells was quantified with real‐time PCR, and IL‐34 expression was significantly inhibited by JQ‐1 (Figure 5E).

### 3.5 IL‐34 overexpression was associated with cancer cell proliferation and chemoresistance

To examine the function of IL‐34 in hepatoblastoma cells, IL‐34 or a control gene was transfected into Huh6 cells, and transfected cells were selected with G418. IL‐34‐producing Huh6^IL34^ and control Huh6^CT^ cells were confirmed with IHC for IL‐34 (Figure 6A). The proliferation of Huh6^IL34^ cells was significantly faster than that of Huh6^CT^ cells (Figure 6B, C). The sensitivity to the anti‐cancer agents, cisplatin (CDDP) and doxorubicin (DXR), was tested, and the IC50 for CDDP was lower in Huh6^IL34^ cells than in Huh6^CT^ cells (Figure 6D). The sensitivity to DXR was not different between Huh6^IL34^ cells and Huh6^CT^ cells.

**IGURE 6:**
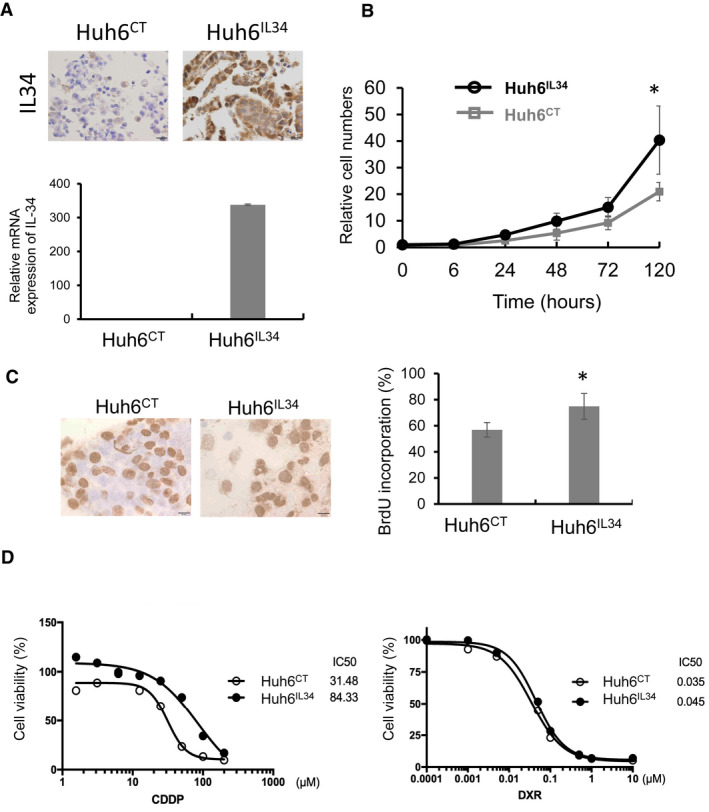
The function of IL‐34 in hepatoblastoma cells. (A) Plasmids encoding the IL‐34 gene and control gene were transfected into Huh6 cells, and selection was performed with G418. IL‐34 expression in Huh6^CT^ and Huh6^IL34^ cells was evaluated with IHC and real‐time PCR. (B) Cell proliferation of Huh6^CT^ and Huh6^IL34^ cells was evaluated with microscopy for 5 days (*n* = 3–4). (C) The BrdU incorporation assay was performed, and the percentage of BrdU‐positive cells was evaluated with IHC. BrdU‐positive cells among 100 randomly selected cells were counted under a microscope (*n* = 3). (D) Huh6^CT^ and Huh6^IL34^ cells were cultured with cisplatin (CDDP) or doxorubicin (DXR) for 48 h, and cell viability was tested with the WST assay. *p‐value <0.05 by the Student's *t*‐test

## 4 DISCUSSION

In the present study, we showed potential protumor functions of CD163‐positive M2‐like macrophages in hepatoblastoma cases. Embryonal components are thought to have higher malignant potential because of their higher Ki‐67 labeling index, and an increased number of M2‐like macrophages had infiltrated into embryonal components. Many articles on the significance of TAMs in malignant tumors have been published, but no studies have investigated TAMs in hepatoblastoma. Therefore, we believe the present study is the first report to describe the significance of TAMs in hepatoblastoma.

In the present study, IL‐6 was suggested to be a growth factor secreted by macrophages that acts on hepatoblastoma cells. Macrophages produce growth factors for cancer cells such as IL‐6, which is critical for hepatocellular carcinoma, via Stat3 activation.^24^, ^25^ Stat3 activation induces many protumor molecules related to cancer cell growth, stem cell properties, immunosuppression, and angiogenesis.^26^, ^27^ Stat3 activation is also involved in the protumor function of macrophages.^28^ CD163 expression induces Stat3 activation,^29^, ^30^ and direct contact between macrophages and glioma cells induces strong CD163 expression in macrophages via Stat3 activation.^31^ CD163 is suggested to be involved in the secretion of protumor cytokines such as IL‐6 in macrophages. The CD163/IL‐6/Stat3 pathway is suggested to be critical in protumor TAMs, however, macrophage‐mediated cancer cell proliferation was not affected by anti‐IL‐6R antibody (unpublished data). Since it is well known that TAMs secrete many protumor factors,^32^ unknown protumor factors derived macrophages might be involved in macrophage‐mediated hepatoblastoma cell growth.

Our in vitro study using hepatoblastoma cell lines and HMDMs showed that direct contact with macrophages induced tumor cell proliferation and Brd4 expression, which induced IL‐34 expression. Brd4 is also a transcription factor for c‐Myc and Bcl2, and Brd4 inhibition induces apoptosis of leukemia cells.^33^ However, the anti‐tumor effects of the Brd4 inhibitor on hepatoblastoma cells were weak in the present study.

In the present study, we also found that IL‐34 and Brd4 expression were specifically detected in embryonal components. Colony‐stimulating factor‐1 (CSF‐1) receptor (CSF‐1R) is a critical molecule that controls the survival, proliferation, and differentiation of TAMs, and IL‐34 was identified as a second ligand for CSF‐1R.^34^ IL‐34 derived from keratinocytes and neurons is involved in Langerhans cells and microglia development.^35^ High IL‐34 expression is correlated with high infiltration of TAMs in the tumor microenvironment, and patients with high IL‐34 expression and high TAM density have a worse prognosis with shorter overall survival and recurrence‐free period.^36^ Chemoresistant lung cancer cells secrete IL‐34, which is associated with immune suppression by inducing the M2‐like phenotype of TAMs,^37^ and co‐expression of IL‐34 and CSF‐1 in cancer cells is correlated with a worse clinical course in patients with lung cancer.^38^ IL‐34 is expressed on several solid cancers including colorectal cancer and hematological malignancies.^39^, ^40^, ^41^, ^42^ Zhou SL and colleagues showed miR‐28‐5p deficiency in hepatocellular carcinoma was closely associated to cancer metastasis by inducing IL‐34 overexpression and increased TAM infiltration.^36^ Cancer‐derived IL‐34 may play a role in resistance to immune checkpoint inhibition therapy.^43^ Consistent with this hypothesis, an anti‐IL34 antibody enhances anti‐PD‐1 therapy in a murine tumor model.^44^ IL‐34 blocking abrogated the TAM infiltration in murine HM‐1 and CT26 tumor model and increased the infiltration of NOS2‐positive M1‐like TAMs,^44^ and this suggested IL‐34 was also involved in M2‐polarization of TAMs. IL‐34 expression was potentially linked to not only cell proliferation and chemoresistance in hepatoblastoma in autocrine manner, but also protumor TME development by inducing TAM infiltration.

**FIGURE 7:**
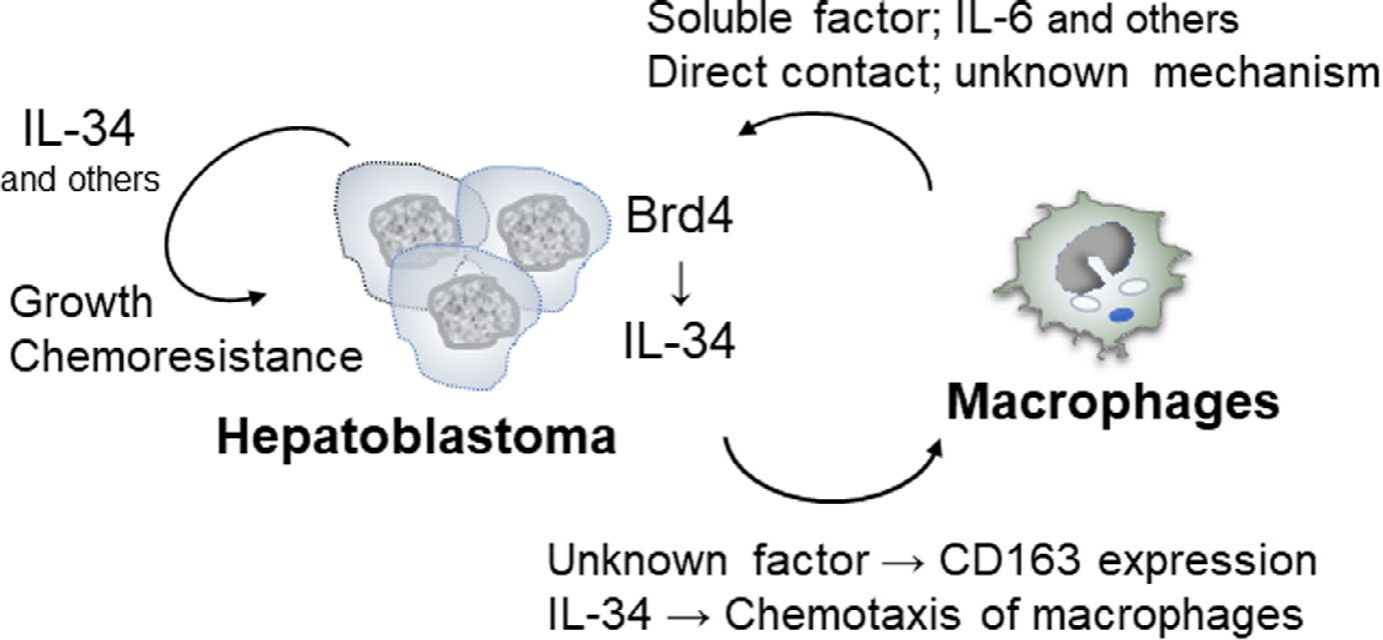
The suggested scheme of cell‐cell interaction of hepatoblastoma cells and macrophages

In conclusion, a high density of CD163‐positive TAMs was seen in embryonal components of hepatoblastoma cases, and Brd4‐induced IL‐34 production was suggested to induce infiltration of TAMs in embryonal components (Figure 7). TAMs may accelerate tumor cell proliferation by secreting protumor cytokines including IL‐6. IL‐34 was also potentially associated with tumor cell proliferation and chemoresistance in an autocrine manner. IL‐34 may be a promising target for anti‐hepatoblastoma therapy. In addition, Brd4 and IL‐34 may be novel markers for embryonal components of hepatoblastoma cases.

## CONFLICT OF INTEREST

This research did not receive any specific grant from funding agencies in the public, commercial, or not‐for‐profit sectors. All authors have no financial competing interests to declare.

## AUTHOR CONTRIBUTIONS

TI, DY, and YK provided study concept and design. YF, MK, MH, SS, TM, KK, YO, and TH participated in the data collection. TI, DY, and YK had full access to all the study data, wrote the initial draft of the manuscript and had the final responsibility for the decision to submit for publication. All authors participated in drafting reviewing and approval of the final manuscript.
